# Road User Exposure from ITS-5.9 GHz Vehicular Connectivity

**DOI:** 10.3390/s22186986

**Published:** 2022-09-15

**Authors:** Martina Benini, Marta Parazzini, Marta Bonato, Silvia Gallucci, Emma Chiaramello, Serena Fiocchi, Gabriella Tognola

**Affiliations:** 1 Department of Electronics, Information and Bioengineering (DEIB), Politecnico di Milano, 20133 Milan, Italy; 2 Institute of Electronics, Computer and Telecommunication Engineering (IEIIT), Consiglio Nazionale delle Ricerche (CNR), 20133 Milano, Italy

**Keywords:** V2X, vehicular connectivity, RF exposure, RF dose assessment, road user

## Abstract

This study addressed an important but not yet thoroughly investigated topic regarding human exposure to radio-frequency electromagnetic fields (RF-EMF) generated by vehicular connectivity. In particular, the study assessed, by means of computational dosimetry, the RF-EMF exposure in road users near a car equipped with vehicle-to-vehicle (V2V) communication antennas. The exposure scenario consisted of a 3D numerical model of a car with two V2V antennas, each fed with 1 W, operating at 5.9 GHz and an adult human model to simulate the road user near the car. The RF-EMF dose absorbed by the human model was calculated as the specific absorption rate (SAR), that is, the RF-EMF power absorbed per unit of mass. The highest SAR was observed in the skin of the head (34.7 mW/kg) and in the eyes (15 mW/kg); the SAR at the torso (including the genitals) and limbs was negligible or much lower than in the head and eyes. The SAR over the whole body was 0.19 mW/kg. The SAR was always well below the limits of human exposure in the 100 kHz–6 GHz band established by the International Commission on Non-Ionizing Radiation Protection (ICNIRP). The proposed approach can be generalized to assess RF-EMF exposure in different conditions by varying the montage/number of V2V antennas and considering human models of different ages.

## 1. Introduction

With one billion and four hundred million vehicles on the road, nowadays, the automotive field is strictly integrated into our society, and the improvement of safety on roads is a timely and important topic. Thus, a lot of research has been conducted on the realization of new wireless vehicular technologies and remote sensing towards the new concept of intelligent transport systems (ITSs). ITSs comprise several services to enable safer roads, such as the so-called vehicle-to-everything communication (V2X). V2X is a vehicular communication technology that entails smart connected vehicles capable of communicating with other entities, such as other vehicles (vehicle-to-vehicle communication, i.e., V2V), pedestrians (vehicle-to-pedestrian communication, i.e., V2P), infrastructures (vehicle-to-infrastructure communication, i.e., V2I), and the network (vehicle-to-network communication, i.e., V2N). V2X communication is mainly based on two wireless access technologies: WiFi for mobility, based on the well-consolidated IEEE 802.11p protocol (or its European version ITS-G5) [1] and the recent cellular technology called Cellular-V2X (C-V2X) [2,3,4,5].

As observed in [6,7], it is expected that vehicular communication will be widespread over the next years. Therefore, the situation will be such that people in a car or its vicinity would be frequently exposed to the radiofrequency electromagnetic field (RF-EMF) generated by such wireless technologies. For this reason, the international standard IEEE/IEC 62704-1 [8] was implemented in 2017 to provide guidelines on the assessment of exposure levels in the human body, whereas the IEEE/IEC 62704-2 [9] specifically addresses the assessment of the exposure generated from antennas mounted on the vehicle.

Most of the studies on vehicular connectivity are typically focused on aspects other than the assessment of RF-EMF exposure, such as the improvement, reliability, and safety of the transmission link in vehicular communications and the optimization of the antenna design (see e.g., [10,11,12]). As for RF exposure in a connected car, a recent literature survey [13] evidenced that most previous studies (see e.g., [14,15,16]) have been focused on the assessment of the exposure to generic personal wireless communication technologies used by the passengers inside the car, such as mobile phones, Bluetooth, and WiFi devices. Only a few studies—references [17,18]—aimed to assess the exposure generated specifically by V2X communication in the car. In particular, the study by Tognola et al. [17] investigated the dose of RF-EMF absorbed by a human model inside a vehicle equipped with V2V antennas operating in the ITS-G5 5.9 GHz band. The other study by Ruddle et al. [18] analyzed the in-vehicle electric field strength generated by a dedicated short-range communication (DSRC) device operating at a frequency of 5.8 GHz for Electronic Toll Collection (ETC), another example of V2X connectivity technology.

Up to now, no studies have investigated RF exposure generated by vehicular connectivity outside the car. Thus, this study aimed to assess, by means of electromagnetic computational techniques, RF exposure in road users (e.g., pedestrians) in the vicinity of a connected car equipped with V2V communication at the ITS-G5 5.9 GHz band.

## 2. Materials and Methods

The present section shows the set-up of the exposure scenario investigated and the methodology used to assess the RF exposure levels on a pedestrian.

### 2.1. The Simulated Exposure Scenario

**Figure 1:**
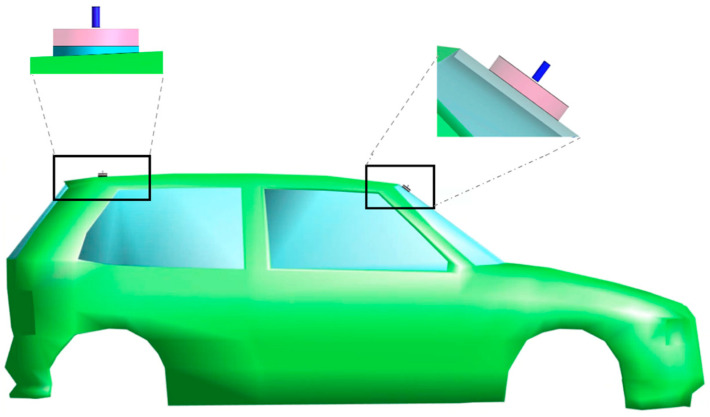
Lateral view of the 3D CAD model of the car with two quarter-wave monopole antennas mounted perpendicularly on the back of the roof and on the windscreen. The radius (R) and height (H) of the ground (pink cylinder) and the arm (blue cylinder) of the two monopoles were chosen so that the antennas resonate at 5.9 GHz, i.e., R = 25 mm, H = 10 mm for the ground and R = 2.3 mm, H = 12 mm for the arm. The model of the antenna at the back also included a substrate of polycarbonate (light blue cylinder) to mimic the effect of the shark-fin case.

The simulated exposure scenario consisted of a human model in the nearby of a car equipped with vertical V2V antennas. Without losing generality and according to the typical installation recommendations [19,20,21], two V2V antennas were used and mounted on the roof at the back of the car and on the windscreen, as seen in Figure 1.

The frontal antenna was perpendicular to the windscreen and, as such, it was tilted to the horizontal plane of 36.5°. The two V2V antennas were modeled as quarter-wave monopoles [17,22] operating at the conventional ITS-G5 5.9 GHz band, as indicated in the IEEE 802.11p protocol [1]. The ground and arm of the antennas were modeled as perfect electric conductor (PEC) material. To simulate as best as possible a realistic scenario, the monopole at the back also included a substrate layer of polycarbonate to replicate the plastic sheet of the typical shark-fin case where the back antenna is placed in real installations [22,23,24]. This substrate had the same geometrical dimension of the ground; the dielectric properties were: density—*ρ* = 1200 kg/m^3^, relative permittivity—ε_r_ = 2.9, and conductivity—σ = 1 × 10^−13^ S/m. Each antenna was fed with a harmonic signal of a reference input power of 1 W (i.e., 30 dBm). As the SAR levels are directly proportional to the input power, the normalization of the exposure levels to 1 W would ease the comparison with further studies that use a different input power. In the air, the reflection coefficient was −17.4 dBi for the back antenna and −20.3 dBi for the front antenna. The antennas’ internal reference resistance was 50 Ω. The two antennas were mounted on a 3D CAD model of a real city car with the dimensions of 1579 (width) × 3814 (length) × 1151 (height) mm. The car model consisted of a body made of PEC and six windows made of glass (*ρ* = 2500 kg/m^3^, ε_r_ = 4.82, and σ = 0.0043 S/m). The interior of the car was filled with air since the electric field generated by an external/internal source is not significantly affected by the materials typically used in the interiors of cars (foam and thin plastic materials) [25].

**Figure 2:**
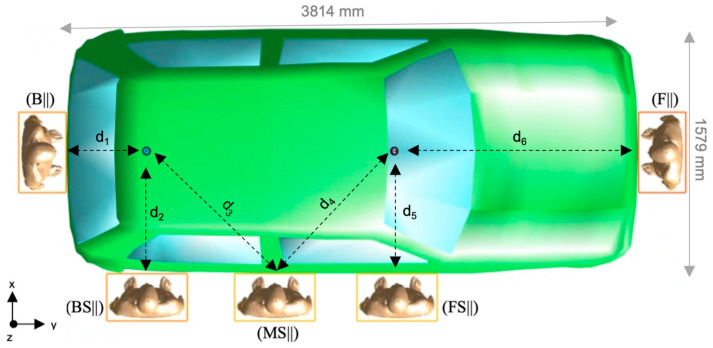

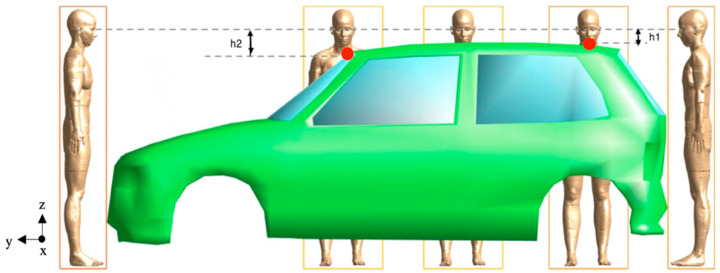
(**Top** panel): Top view of the human model in the front (F||), back (B||), front-side (FS||), middle-side (MS||), and back-side (BS||) configuration. This picture also displays the dimensions of the car and the distances “d” between the Huygens’ Box and the antennas: d_1_ = 536 mm, d_2_ = d_5_ = 815 mm, d_3_ = d_4_ = 1179 mm, d_6_ = 1608 mm. (**Bottom** panel): Lateral view of the car and the human model in the different configurations. The relative height between the eyes and the rear antenna is h_1_ = 80 mm and that between the eyes and the front antenna is h_2_ = 155 mm. The yellow rectangle is the “Huygens’ Box” used in the FDTD method to calculate the dose of the EMF absorbed by the human model (see the details in Section 2.2, “Electromagnetic field calculation”).

**Figure 3:**
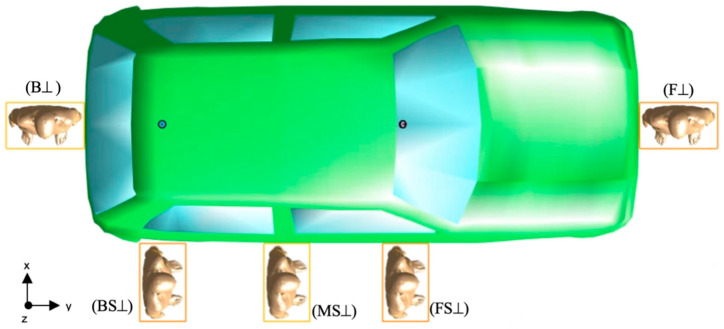
Top view of the human model in the front (F⊥), back (B⊥), front-side (FS⊥), middle-side (MS⊥), and back-side (BS⊥) configurations. The distances between the Huygens’ Box (yellow rectangle) and the antennas are the same as in Figure 2 (top panel).

The human model “Ella”, a 26-year-old girl (height = 1.63 m, weight = 57.3 kg, BMI = 21.6 kg/m) from the virtual population VIP4.0 was considered (https://itis.swiss/virtual-population/virtual-population/overview/ (accessed on 26 July 2022)). The dielectric properties of the human model’s tissues at 5.9 GHz were set according to the literature data [26]. According to the technical standard IEEE/IEC 62704-2 [9], to assess how the absorption of the RF-EMF varied with distance from the antennas, the human model was placed at five different positions next to the car, namely at the front (F), back (B), front-side (FS), middle-side (MS), and the back-side (BS) of the car. For each of these five positions, we considered two orientations—frontal (||) and perpendicular (⊥)—of the human model relative to the body car, thus resulting in ten different configurations, as seen in Figure 2 and Figure 3.

### 2.2. Electromagnetic Field Calculation

The EMF generated by the two antennas in the simulated scenario was computed using the finite-difference time-domain (FDTD) method, as implemented in the Sim4Life platform [27]. The FDTD is an iterative numerical approach used in computational bioelectromagnetics to calculate the EMF in a simulated exposure scenario that is modeled using 3D geometries; the scenario is then discretized in terms of grids, and Maxwell’s equations are solved at each point in the grid.

In our simulations, the FDTD computational domain contained the model of the car, the two antennas, and the human model placed near the car in one of the ten configurations at a time, thus giving rise to ten different EMF simulations. In each simulation, the computational domain was discretized with a non-uniform grid with a maximum step of 1 mm for the back antenna and 0.2 mm for the front antenna. The front antenna was discretized with a step smaller than the back antenna to reproduce as best as possible its tilted shape. The tissues of the human model were discretized with a maximum step of λ/10 [27] (where λ = *c*/(*f* · *√ε_r_*) is the wavelength (m), *c* is the speed of light (m/s), *f* is the wave frequency (Hz), and *ε_r_* is the relative permittivity), depending on the tissues’ dielectric properties. For example, the discretization step was 0.86 mm for the skin and 0.632 mm for the eye tissues (i.e., the cornea, eye sclera, eyes vitreous humor, and eye lens). The simulation duration was set to 80 periods with a time step of 0.16 ps to reach a steady state condition; the convergence level of the FDTD iterative procedure was set at −120 dBm. Furthermore, the domain was truncated assuming a perfectly matched layer (PML) absorbing condition at the domain boundaries.

The volume of the computational domain ranged from 20.6 m^3^ to 29.3 m^3^ depending on the considered exposure scenario configuration. We used the Huygens’ Box approach [28,29] to reduce the overall computational time [30]. This approach is based on the electromagnetic equivalent principle, which states that the EMF generated inside a region by sources external to the region is the same as that generated by equivalent surface currents on the region boundary called “Huygens’ Box”. In our simulations, the Huygens’ Box (see the yellow rectangles in Figure 2 and Figure 3) delimitated a 3D rectangular volume of 536 mm × 316 mm × 1666 mm size around the human model. Thanks to the equivalent principle, the EMF computation in the simulated exposure scenario could be split into two subsequent stages:

1. In the first stage, the simulation was done without the human model and aimed to calculate the equivalent electric and magnetic surface currents on the Huygens’ Box, defined as $\vec{J_{s}}=\hat{n}\times \vec{H}$ and $\vec{M_{s}}=\hat{n}\times \vec{E}$, where $\vec{n}$ is the inward-pointing normal vector to the Huygens’ Box and $\vec{H}$ and $\vec{E}$ are the magnetic and electric fields inside the Huygens’ Box due to the two antennas on the car;
2. In the second stage, the human model was placed inside the Huygens’ Box region and the equivalent surface currents calculated in the first stage were used as the excitation sources, i.e., they acted as sources generating the same fields as those originated by the two antennas.

As demonstrated in [30], the combination of the FDTD method with the Huygens’ Box approach provides high numerical accuracy and many benefits for the calculation time. In [30] the authors applied the Huygens’ Box approach to calculate the SAR distribution around a pacemaker worn by an adult male phantom inside an RF magnetic resonance imaging (MRI) birdcage. They pointed out (i) the accuracy of this approach and (ii) the 8-fold factor reduction in the simulation time and the less RAM memory required for the calculation.

### 2.3. Exposure Assessment

We assessed the exposure of the human model to the EMF generated by the two antennas by analyzing (*i*) the specific absorption rate (SAR) of the EMF over the whole body (wbSAR), defined as the ratio between the power of the EMF absorbed by the whole body and the total mass of the body (W/kg), (*ii*) the SAR averaged over 10 g of tissue (SAR_10g_), and (*iii*) its peak value (pSAR_10g_) for the 10 different exposure configurations of the human model near the car. Since the EMF does not penetrate deep into tissues at 5.9 GHz [31,32], we focused our analysis on the most superficial tissues and organs, namely the skin and the eyes. More specifically, we investigated the SAR in the skin of the whole body, locally at the head and genital areas, and in the cornea, sclera, lens, and vitreous humor. To quantify and compare the dispersion of the distributions of the SAR across tissues and human model configurations, we computed the quartile coefficient of dispersion (QCD), defined as:

QCD = (Q_3_ − Q_1_)/(Q_1_ + Q_3_), (1)

where Q_1_ and Q_3_ are the 25th and 75th quartiles of the SAR distribution. Finally, to investigate the spread of the SAR around its maximum, we also computed P_≥0.70 · pSAR10g_ which corresponds to the percentage of SAR_10g_ values greater than 0.7·pSAR_10g_. P_≥0.70 · pSAR10g_ was analyzed to evaluate if the points with a high SAR_10g_ value were localized in narrow or broad regions around the maximum. Please note that the value of 0.7·pSAR_10g_ corresponds to a SAR_10g_ value of −3 dB of the maximum.

## 3. Results

We remind the readers that all the values reported in this section were obtained by considering an input power of 1 W (i.e., 30 dBm) for each V2V antenna and considering both of the antennas switched on, as described in the Materials and Methods.

Table 1 lists the wbSAR values across the examined human model configurations. It was observed that the wbSAR in the frontal orientation was always greater than in the perpendicular one. Furthermore, in both orientations, the highest wbSAR (equal to 0.19 and 0.12 mW/kg for the frontal and perpendicular orientation, respectively) was observed when the human model was in position B, which is nearest to the back antenna. In all the positions and orientations, the wbSAR was well below the basic restriction limit of exposure for the general population in the 100 kHz–6 GHz range of 0.08 W/kg, as set by the ICNIRP [33] and IEEE [34].

**Figure 4:**
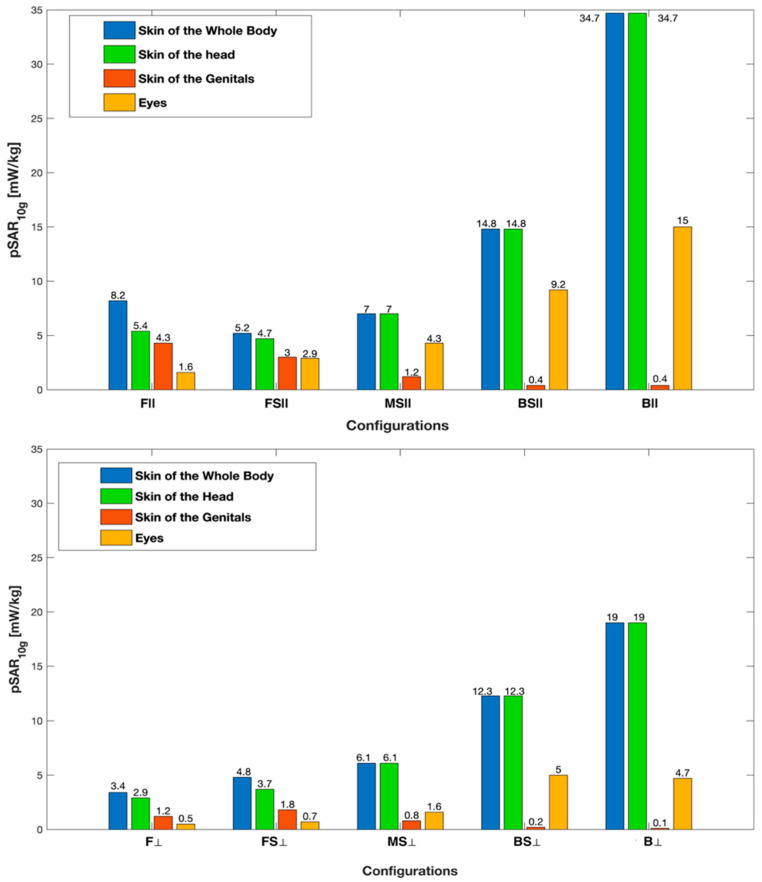
pSAR_10g_ across tissues as a function of the human model configuration in the frontal (||, **top** panel) and perpendicular (⊥, **bottom** panel) orientations.

In Figure 4, pSAR_10g_ in each examined tissue is shown as a function of the human model position and orientation. The maximum value of pSAR_10g_ (equal to 34.67 mW/kg) was in the skin of the head, in position B, that is, when the human model was facing the back of the car. Indeed, this was the configuration in which the human model was closest to one of the two antennas (the rear one), at a distance of d_1_ = 536 mm (Figure 2, top panel).

As we observed for the wbSAR, pSAR_10g_ in Figure 4 was greater in the frontal orientation than in the perpendicular one. In addition, in all the configurations, pSAR_10g_ was greater in the skin than in the eyes, and within the skin, the head region always had greater pSAR_10g_ values than the genital area. It is interesting to note that pSAR_10g_ in the upper body (i.e., in the head and eyes) gradually decreased from its maximum in position B to its minimum in position F. This is because the distance between the antenna and the human model progressively increased from position B to F (see Figure 2, top panel). pSAR_10g_ in the skin at the genital area was generally negligible except in positions F and FS, where the field radiated from the tilted frontal antenna hits the lower body in addition to the head and eyes.

**Figure 5:**
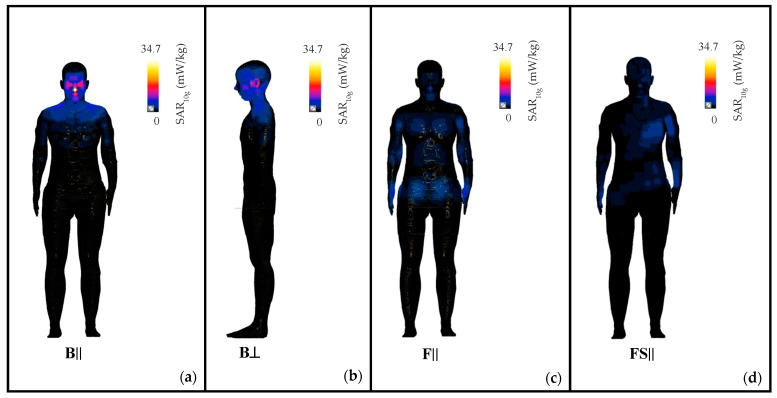
Distribution of the SAR_10g_ over the skin of the whole body in configurations B|| (**a**) and B⊥ (**b**), which corresponded to the exposure scenario with the highest exposure level in the head region, and in configurations F|| (**c**) and FS|| (**d**), which corresponded to the exposure scenarios with the highest exposure level for the genital region. In all the cases, the SAR_10g_ was normalized to the maximum found in the worst case (i.e., in the B|| configuration), which was equal to 34.7 mW/kg.

To further explore how the RF-EMF dose is absorbed in the skin, which is the body tissue with the greatest exposure level, Figure 5 shows the SAR_10g_ distribution on the skin of the human model in positions B, F, and FS. Indeed, according to our results (see Figure 4), in position B there is the maximum exposure for the upper-body region (i.e., the head region) and in the F and FS there are the maximum exposures for the lower-body region (i.e., the genital area). Figure 5 shows that in position B, the exposure at the head region was localized at the nose and eyelids for the B|| configuration (Figure 5a) and at the skin of the ear for the B⊥ configuration (Figure 5b). This means that the exposure was localized at those body parts closest to the rear antenna. Vice versa, in the same position B, the SAR_10g_ in the torso and limbs was negligible. In positions F and FS (Figure 5c and Figure 5d, respectively), the exposure was localized not only at the head but also in the lower body regions, i.e., the limbs, the hands, and the genitals. This is probably because in positions F and FS, the frontal antenna, being tilted more downward than the back antenna, generated a field that radiated to the lower and the upper body.

**Figure 6:**
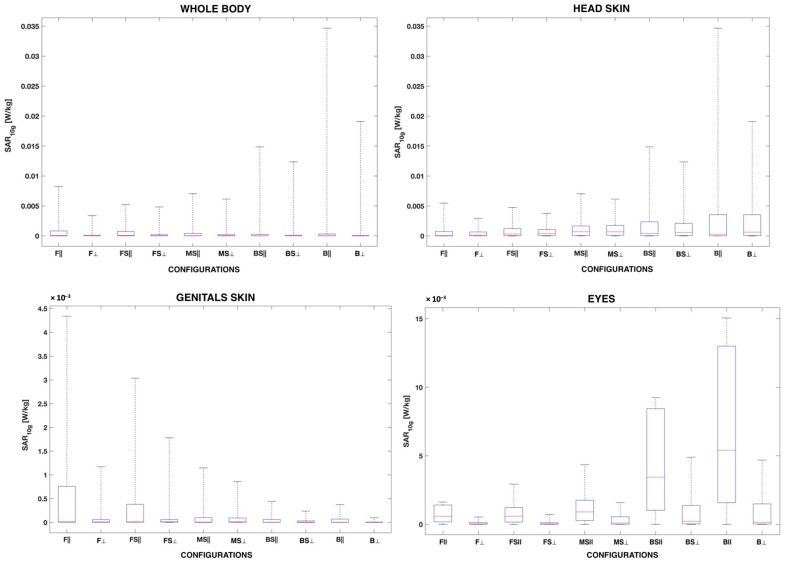
Box plot of the SAR_10g_ distribution in the skin of the whole body, head, and genitals and the eye tissues as a function of the human model configuration. The red mark in the center of the box indicates the median; the bottom and the top edges of the box indicate the 25th and 75th percentiles, respectively. The upper and lower whiskers represent the maximum and minimum SAR_10g_ values.

Figure 6 compares the distribution of the SAR_10g_ across the human model configuration in the skin of the head and genitals, and the eye tissues.

As a general comment, it can be seen in Figure 6 that in all the tissues and configurations, the SAR_10g_ distribution was positively skewed, meaning that the SAR_10g_ was mainly distributed towards low exposure levels and that only a few samples in each tissue were characterized by high SAR_10g_ values. For all the tissues except the eyes, the median SAR_10g_ value was generally very low across all human model configurations, especially when the SAR was evaluated over wider areas of the body, such as the skin of the whole body. Differently from the other tissues, in the eyes, the median SAR_10g_ varied with the orientation of the human model, being greater when the human model was in the frontal orientation rather than in the perpendicular one.

The QCD was generally high and ranged from 0.72 to 0.99 across tissues and human model configurations, which indicates that the SAR_10g_ distribution had a high degree of dispersion/variability within each tissue. Among the tissues, the QCD of the skin (considering the whole body, head, and genitals) was higher than for the eyes, being equal to 0.88–0.99 for the skin and 0.72–0.98 for the eyes, meaning that the dispersion (that is, the variability) of the SAR_10g_ was greater in the skin than in the eyes.

To have a clearer view of the spread of the SAR_10g_ around its maximum, Table 2 lists the values of P_≥0.7 · pSAR10g_ across the examined configurations for the eyes and the skin at the genital and head areas. As shown in Table 2, among all the tissues, the highest P_≥0.7 · pSAR10g_, that is, the wider spatial spread, was observed at the eyes in the BS|| configuration. In this latter configuration, P_≥0.7 · pSAR10g_ was at a maximum of 33.6%, meaning that more than one-third of the eye tissues were characterized by high SAR_10g_ values. The spread of the SAR_10g_ observed in the remaining tissues was lower and had a maximum of 6.3% in the skin of the genitals in the F|| configuration and 1.5% in the skin of the head in the MS|| configuration. It is worth noting that the skin of the head had the smallest P_≥0.7 · pSAR10g_, meaning that the EMF exposure in this tissue was much more localized in a narrower region than in the other tissues.

## 4. Discussion

This study presents an assessment of the RF-EMF exposure of a road user in a vehicular connectivity exposure scenario, which, to the best knowledge of the authors, has never been studied before.

The maximum values of the whole body and the local SAR calculated in the present study across the different positions and orientations of the human model near the car were 0.19 mW/kg for the whole body, 34.7 mW/kg in the skin of the head, and 15 mW/kg in the eyes. In all cases, the exposure levels were well below the basic restriction limits set by the ICNIRP [33] and IEEE guidelines [34] for the general public in the 100 kHz–6 GHz band, that is 0.08 W/kg for the whole body and 2 W/kg in 10 g of tissue of the head and torso regions. As indicated by the technical specification of IEEE 802.11p [1], the maximum transmitted power of the V2X communication antennas is 33 dBm in the EU and the US, reaching 44.8 dBm in the US for government services. If, in our simulations, the input power of the two V2X antennas was scaled to the maximum transmitted power of 33 dBm each, i.e., 1.99 W, the levels of exposure would be almost doubled. It is interesting to note that also in this case, the exposure levels would remain well below the limits imposed by the ICNIRP [33] and IEEE guidelines [34]. The same exposure levels would remain below the basic restriction [33,34] even if the input power for each antenna was scaled to 44.8 dBm, i.e., 30 W.

In all the configurations, the RF dose was mainly absorbed in the most superficial tissues (i.e., the skin) and organs (i.e., the eyes). Our results show higher SAR values in the frontal orientation, i.e., when the human model was facing the antennas. Among all the configurations, the highest pSAR_10g_, equal to 34.67 mW/Kg, was observed in the skin of the head in the B|| configuration, i.e., when the human model stood at the back of the car. It is worth noting that, in the layout we modeled, the B|| configuration was the one that corresponded to the shortest distance between the antenna (the back one) and the human model.

In all the configurations, the RF power was absorbed mainly in the skin of the head and at the eyes because the head/eye is the body region closer to and at the same height as the antennas. On the contrary, the skin of the genitals always had negligible values of the SAR except in the F and FS positions, that is, when the phantom stood closer to the front antenna. This was because, in our simulated scenario, the frontal antenna was tilted further downward than the back antenna, and thus, its field could also reach the lower body regions together with the upper regions of the body.

The analysis of P_≥0.7 · pSAR10g_ showed that the spread of the SAR distribution differed among the examined tissues. Namely, for the head and the genitals, the exposure was localized in a narrow area, corresponding to maximums of 1.5% and 6.3% of the skin of the head and genital areas, respectively, whereas in the eyes, the region with high exposure extended to up to 33.6% of the eye tissues. Furthermore, regarding the head, we found the highest spread (1.5%) in the MS|| configuration, that is, when the human model stood at the middle side of the car because, in this position, the head was exposed to the left and right antennas simultaneously.

As a general remark, the distribution of the SAR_10g_ for all tissues and configurations showed a positive skewness, meaning that the SAR_10g_ is distributed mainly in the lower range of exposure levels. In the eyes, the SAR_10g_ distribution was less skewed than in the other examined tissues, especially when the human model was in the frontal orientation.

No studies have assessed the RF-EMF exposure in people *outside* a car equipped with V2V antennas, as we did in the current study. As anticipated in the Introduction, there have only been two studies—references [17,18]—that have investigated the exposure to RF-EMF generated by vehicular communication inside the car. Below is a qualitative comparison of our results with those of [17,18].

Tognola et al. [17] investigated the SAR in a passenger (a driver) inside a car equipped with the same V2V antennas used in this study. The authors of [17] mounted four antennas with a symmetric montage at the front/rear side of the car roof and the left/right mirrors. They also investigated an additional worst-case “asymmetric montage”, where all the antennas were switched on, and the front and left antennas were moved closer to the head than in the symmetric montage. Each antenna was operated at the maximum power allowed by the IEEE 802.11p protocol, i.e., at 44.8 dBm (30 W). The highest wbSAR found by [17] in such worst-case conditions was 8.33 mW/kg. If we scale our results by feeding each of the two V2V antennas of our scenario with the maximum input power of 30 W, the wbSAR in our highest exposure configuration (i.e., in the B|| configuration) would be 5.7 mW/kg. The wbSAR in [17] was greater than in our worst case, most probably because of the higher number of antennas used in [17] (four antennas in [17] instead of two antennas). Furthermore, this difference may also be due to the shorter antenna–head distance in [17] compared to our scenario, even if in [17], the PEC material of the chassis would mitigate the exposure of the passenger inside the car; whereas, in our scenario, there was direct exposure between the antenna and the head. As for the local SAR, the highest value found in [17] was at the head, as in our study. In particular, in [17], the SAR in the head was 1.581 W/kg in the “asymmetric montage”, which is greater than the 1.040 W/kg we would have obtained in the B|| configuration if our antennas were operated at the maximum input power of 30 W. Again, it is possible that the SAR we observed at the head was lower than in [17] because, while in our scenario, only the rear antenna was close to the head (at a distance of about 0.5 m), in the “asymmetric montage” of [17], both the front and left antennas were close to the head, at a distance of 0.1 m for the frontal antenna and less than 0.5 m for the left antenna. This resulted in a higher exposure level in the head region in the scenario evaluated in [17].

As a last remark, it is worth noting that the exposure scenarios addressed in [17] and in our study are different not only for what concerns the number of antennas used simultaneously but also for the different durations of typical exposure, which is expected to be longer for a passenger in the connected car than for the pedestrian.

The authors of [18] estimated the power density generated inside a vehicle by an external DSRC device at 5 m from the car, operating at 5.8 GHz. Inside the vehicle, they found a power density of 0.00637 W/m^2^. In our scenarios, in the vicinity of the pedestrian, the power density was lower than in [18] and equal to 0.023–0.22 W/m^2^. This is because our antennas were much closer than 5 m from the human model. However, our values are below the limits of 10 W/m^2^ set by ICNIRP [31] for the general public in the 2–300 GHz.

## 5. Conclusions

In this study, we investigated, for the first time, the dose absorbed by a pedestrian in a V2V outdoor exposure scenario by means of a deterministic numerical dosimetry approach. We analyzed five different positions of a pedestrian close to the car in two different orientations. The SAR varied with the positions and orientations of the pedestrian near the car. We did not find SAR levels higher than those with a potentially adverse effect on human health in any case. The frontal orientation (i.e., when the human model was facing the antennas) always resulted in higher levels of SAR compared to the perpendicular one. We further identified the case with the highest exposure as that when the human model stood close to the back antenna because, in this position, the distance between the human model and the antenna was the shortest. The tissue much affected by higher levels of exposure was the skin of the head.

The proposed approach can be generalized to assess the RF-EMF exposure in vehicular communication in different conditions, e.g., by varying the montage and number of V2V antennas and by using human models of different ages, e.g., children, neonates, and pregnant women. Moreover, to fully assess the SAR levels induced by vehicular connectivity, the stochastic dosimetry [35] and machine learning [36] approaches could be applied to further address the variability of the exposure scenario. Finally, further studies should be conducted to assess the influence that the outdoor environment (such as the presence of buildings, trees, and the scattering of other vehicles in the streets) might have on the exposure level.

## Figures and Tables

**Table 1:** The wbSAR across human model positions in the frontal and perpendicular orientations.

Position Near the Car	wbSAR (mW/kg) in the Frontal Orientation (||)	wbSAR (mW/kg) in the Perpendicular Orientation (⊥)
B	0.19	0.12
BS	0.11	0.086
MS	0.11	0.08
FS	0.16	0.086
F	0.17	0.062

**Table 2:** P_≥0.7 · pSAR10g_ for the eyes and the skin at the genitals and head areas across the 10 configurations of the human model. The red text highlights the highest P_≥0.7 · pSAR10g_ values for each tissue.

	P_≥0.7 ·pSAR10g_		
Configuration	Eyes	Genitals Skin	Head Skin
F||	32.9%	**6.3%**	0.2%
F⊥	11.9%	2.5%	0.5%
FS||	17.5%	0.6%	0.3%
FS⊥	7.3%	1.0%	0.2%
MS||	12.8%	2.8%	**1.5%**
MS⊥	16.1%	0.7%	0.7%
BS||	**33.6%**	1.6%	0.2%
BS⊥	12.8%	2.3%	0.3%
B||	32.5%	1.5%	0.2%
B⊥	14.6%	1.4%	0.2%

## Data Availability

Not applicable.
